# Regulatory mechanism and therapeutic potentials of naringin against inflammatory disorders

**DOI:** 10.1016/j.heliyon.2024.e24619

**Published:** 2024-01-18

**Authors:** Yuan Peng, Ruyi Qu, Shuqin Xu, Hongsheng Bi, Dadong Guo

**Affiliations:** a Shandong University of Traditional Chinese Medicine, Jinan, 250002, China; b Affiliated Eye Hospital of Shandong University of Traditional Chinese Medicine, Jinan, 250002, China; c Shandong Provincial Key Laboratory of Integrated Traditional Chinese and Western Medicine for Prevention and Therapy of Ocular Diseases, Shandong Academy of Eye Disease Prevention and Therapy, Medical College of Optometry and Ophthalmology, Shandong University of Traditional Chinese Medicine, Jinan, 250002, China

**Keywords:** Naringin, Inflammatory disease, Anti-Inflammatory, Antioxidant

## Abstract

Naringin is a natural flavonoid with therapeutic properties found in citrus fruits and an active natural product from herbal plants. Naringin has become a focus of attention in recent years because of its ability to actively participate in the body's immune response and maintain the integrity of the immune barrier. This review aims to elucidate the mechanism of action and therapeutic efficacy of naringin in various inflammatory diseases and to provide a valuable reference for further research in this field. The review provided the chemical structure, bioavailability, pharmacological properties, and pharmacokinetics of naringin and found that naringin has good therapeutic potential for inflammatory diseases, exerting anti-inflammatory, anti-apoptotic, anti-oxidative stress, anti-ulcerative and detoxifying effects in the disease. Moreover, we found that the great advantage of naringin treatment is that it is safe and can even alleviate the toxic side effects associated with some of the other drugs, which may become a highlight of naringin research. Naringin, an active natural product, plays a significant role in systemic diseases' anti-inflammatory and antioxidant regulation through various signaling pathways and molecular mechanisms.

## Graphical abstract

**Figure:**
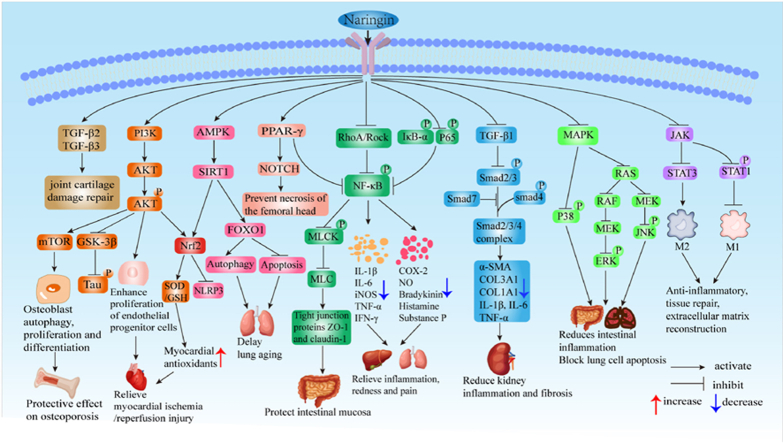

## 1 Introduction

Naringin is a natural flavonoid in grapes, citrus fruits, and Chinese herbal medicine. It is a flavanone glycoside formed by the flavanone naringenin and the disaccharide neohesperidose, mainly derived from yellowish dihydro flavonoids extracted from the dried peel of the plant Rutaceae and grapefruit [1,2]. Isolation of naringin from plants or fruits involves four steps: extraction, identification, isolation, and purification. It is based on microwave-assisted extraction (MAE), high-performance liquid chromatography-photodiode array detector-mass spectrometry (HPLC-DAD-MS/MS), and high-speed countercurrent chromatography (HSCCC) highly effective strategies [3]. Determination of naringin is most commonly performed by high-performance liquid chromatography (HPLC) and ultra-performance liquid chromatography (UHPLC) combined with a mass spectrometer (MS) or photodiode array (PAD) detectors [4]. Studies have found that naringin plays an essential role in promoting anti-inflammatory [5], anti-apoptotic [6], anti-tumor [7,8], anti-ulcer [9], oxidative stress [10], antiviral [11] and promoting skeletal muscle fiber remodeling [12,13]. Naringin has been approved as a potential antitussive and expectorant agent in clinical trials [14]. The powerful anti-inflammatory effect of naringin can treat and relieve a variety of inflammatory diseases in multiple organs of the body and airway inflammation [15], exhibit renal and neuroprotective effects [16,17], and therapeutic effects in the prevention and treatment of cardiovascular disease [18] and metabolic syndrome [19].

## 2 Materials and methodology

A search strategy was performed to extract the available literature from the PubMed database. The search terms “naringin," “bioavailability," “signaling pathways," and “Pharmacokinetics" combined with terms like inflammatory diseases such as neuroinflammatory diseases, cardiovascular inflammatory diseases, inflammatory bowel diseases, renal inflammation, skin inflammation, and joint inflammation were searched. Original research, including prospective and retrospective studies and review papers, was included and cross-referenced.

## 3 Results and discussion

### 3.1 Naringin chemical structure and bioavailability

**Fig. 1:**
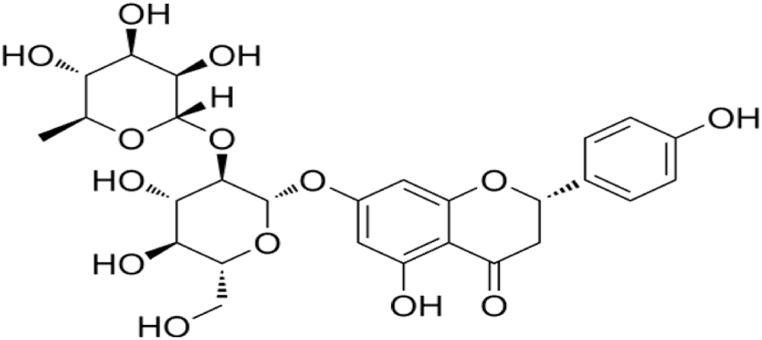
Chemical structure of naringin.

Naringin, chemically 4′,5,7-trihydroxyflavanone-7-rhamnoglucoside (Fig. 1), is weakly alkaline, pale yellow or white powder with the bitter taste of grapefruit juice [20]. Naringenin is the aglycone of naringin, and various biological enzymes can help eliminate the glycosides of naringin to produce naringenin, which is then converted to other metabolites [21,22]. Both naringenin and naringin have anti-inflammatory effects [23]. Naringin is a naringenin, an aglycone and neohesperidose flavanone glycoside bound to the –OH group at the carbon C-7, and it has a bitter taste [20].

Naringin is a natural product with abundant sources, a wide range of pharmacological effects, low toxicity, and few systemic adverse reactions when used as a therapeutic agent [24,25]. The oral bioavailability of naringin in humans has been reported to be only 5–9% [26]. The lower bioavailability may be closely related to the extensive metabolism of naringin, first-pass elimination, and the exocytosis of metabolites [27]. Naringin metabolism is a complex process catalyzed by various enzymes [21,22,28,29]. Phase II enzymes metabolize naringin in the intestine, and the resulting hydrophilic phase II metabolites are transported out of the cell into the intestinal lumen or bile by efflux transporter proteins. Some naringin can be transferred to the liver via enterohepatic circulation and conjugated into glucuronidated and sulfated metabolites that can be absorbed by organs and tissues [27]. There is substantial evidence that naringin has powerful biological activities, and relative safety does benefit human health. Therefore, to improve the bioavailability and pharmacological properties of naringin, it is currently proposed to encapsulate naringin within its structure and thus release it in a controlled manner by using various modern nanocarriers or liposomes such as preparations, including nanogels, phytocomplexes, lipid nanoparticles, polymers, inorganic nanoparticles, and demonstrated that naringin esterification could improve the physical and chemical properties of naringin and thus enhance its bioavailability [[30], [31], [32], [33]]. In vitro and in vivo toxicological evaluation of naringin-loaded nanocapsules and nanocapsules showed no signs of toxicity [34], and the carrier binding to naringin may exert a synergistic effect. The metal-organic framework (MOF) has been shown to encapsulate naringin as a nontoxic loading carrier that can improve and modulate immune system function using the synergy between naringin and MOF [24]. In addition, the naringin-loaded proposomal gel (PPG) improved the permeability of naringin and showed better wound closure [35]. Although naringin has low solubility in lipophilic systems, esterification can overcome it. Lee J et al. have demonstrated that naringenin esterification can improve the physical and chemical properties of naringin and thus enhance its bioavailability [36]. Naringin acetate includes an enzymatic reaction between naringin and an acyl donor, and studies in recent years have confirmed that naringin acetate can promote esterification reactions, resulting in high conversion rates in a short period [37].

### 3.2 Spectral properties of naringin

Citrus flavonoids are polyphenolic flavonoids that exist mainly as glycosides in citrus fruits, with naringin and hesperidin being the most abundant [38]. They can be converted to their glycosidic ligands, naringenin, and hesperidin. To date, there are only scattered fragments of spectroscopic literature on flavonoids. The main reason is the difficulty in obtaining and isolating individual flavonoids and their wide variety of species. Flavonoids are categorized into six groups: isoflavones, flavanones, flavonols, flavonoids, flavanols, and anthocyanins [39]. Naringin, naringenin, hesperetin, and hesperidin are included in the flavanones.

Flavonoids have a similar backbone, with a core structure consisting of an oxygen-containing heterocyclic ring of 15 carbon atoms in a C6–C3–C6 skeleton, represented by rings A, C, and B, respectively [39]. The various flavonoids differ mainly in the different substitution positions of the three groups (hydroxyl, methoxy, and glycosyl). Spectroscopic techniques are an excellent way to identify and compare the structures of various flavonoids.

Surface-enhanced Raman spectroscopy (SERS) has the characteristics of fast analysis, high sensitivity, and non-destructiveness. SERS showed that most citrus flavonoids displayed similar spectra below 1000 cm^−1^ [40]. The characteristic peaks were mainly at 1100-1500 cm^−1^ (assigned to different O–H bond) and 1550-1650 cm^−1^ (assigned to C=O bond) [40]. Among them, the band at 1650-1700 cm^−1^ (assigned to C(H)–C(H) bond) can distinguish flavanones from other analogs [40]. For flavanone analogs, the band at 1221 cm^−1^ belonging to v (C–H) of CH3 can be used to distinguish the hesperetin analogs (hesperidin, hesperetin) from the naringenin analogs (naringin, naringenin) [40]. However, it is challenging to determine naringin from naringenin by using Sers alone, and SERS is now primarily used in combination with thin-layer liquid chromatography for further differentiation.

In addition, naringin and naringenin have slightly different UV absorption properties. The absorption spectra of flavonoids usually consist of two main bands, band I (300–380 nm) and band II (240–295 nm). The band II (283 nm) of naringin was shifted downward by 5 nm compared to naringenin (288 nm), while the band I of naringin and naringenin were relatively unchanged and almost identical [41].

In addition, the typical bands for naringenin, such as carbonyl stretching at around 1660 cm^−1^ for both IR and Raman spectra or vibration of C–C in aromatic ring around 1618 cm^−1^ and 1019 cm^−1^ for IR and at about 1613 cm^−1^ and 1104 cm^−1^ for Raman spectra are present [39]. Thus, the different positions of the three substituents on flavonoids cause differences in spectral features, and joint evaluation of multiple spectroscopic techniques can help to distinguish flavonoids better.

### 3.3 Pharmacokinetics of naringin

Interestingly, there are significant sex-related and species-specific differences in the pharmacokinetics of naringin and naringenin [42]. In trials combining non-clinical and clinical data, the pharmacokinetics and metabolism of naringin after oral and intravenous administration in rats, dogs, and humans were evaluated. It was found that the pharmacokinetic process of naringin was prolonged in humans after oral administration of naringin compared to those in rats and dogs [43], a difference that is thought to be closely related to biochemical processes, blood flow rate, and volume or surface area of the species [43]. After a single oral administration of naringin in a constructed rat animal model, naringin was widely distributed in multiple organs, and the concentration of naringin in tissues was much higher than in plasma, particularly in the trachea and lungs [ [44]， [45]]. In clinical studies, plasma naringin concentrations increased to a maximum at approximately 2 h (Tmax, 2.09 ± 1.15 h) and decreased to 50 % of Cmax at about 3 h (t 1/2, 2.69 ± 1.77 h) [43].

### 3.4 Molecular mechanism of naringin reducing drug toxicity

Naringin has potential hepatoprotective effects on acetaminophen (APAP)-induced injury. The major cellular processes of APAP-induced hepatocytotoxicity include increased oxidative stress and mitochondrial dysfunction [46,47]. Nuclear factor erythroid 2-related factor 2 (Nrf2) is a transcription factor in oxidative stress that regulates cellular defenses against toxicity and oxidative damage by expressing genes for oxidative stress and drug detoxification, and Nrf2 can also participate in metabolic reprogramming, immune response, autophagy regulation, protein balance, inflammation regulation, and mitochondrial physiology regulation [[48], [49], [50]]. In an in vitro model established by primary rat hepatocytes and HepG2 cells, naringin could activate the AMPK/Nrf2 pathway, reducing in vitro oxidative stress by promoting AMPK phosphorylation and upregulating Nrf2 expression [51]. At the same time, this process can promote mitochondrial fusion by increasing the expression of fusion proteins (i.e., Mfn1, Opa1), thereby reducing APAP-induced hepatocellular and mitochondrial damage [51,52]. Similarly, in APAP-induced mouse models of acute hepatotoxicity, naringin mediates Nrf2 activation by upregulating cation transport regulator-like protein 2 (CHAC2), alleviating acetaminophen-induced acute liver injury [53]. In most cases, Nrf2 activation plays a protective role in maintaining human health, and naringin can activate Nrf2 signaling to protect human health. Therefore, naringin plays a role in treating APAP-induced liver injury in mice, reducing the expression of liver injury markers in a dose-dependent manner, with potential mechanisms including antioxidant, rescue glucose metabolism, amino acid regulation, and purine metabolism [54].

Cyclophosphamide (CYCP) is a synthetic alkylated anti-tumor drug that destroys cancer and non-cancer cells, leading to cancer regression and multi-organ toxicity. Naringin can prevent cyclophosphamide-induced hepatotoxicity in rats by attenuating oxidative stress, fibrosis, and inflammation by the molecular mechanism that naringin can attenuate CYCP-induced upregulation of hepatic chemokine ligand 2 (CCL2), interferon (IFN) α1, interleukin (IL) -1β, and transforming growth factor (TGF) β1, reversing CYCP-induced reduction in hepatic glutathione (GSH) levels and catalase (CAT) activity [55]. Naringin exerts its anti-inflammatory, antioxidant, and apoptotic activities by increasing tumor necrosis factor (TNF)-α and IL-17 levels and reducing hepatic p53 and caspase-3 expression, and also has a specific mitigating effect on diclofenac-induced hepatotoxicity in male Wistar rats [56].

Methotrexate (MTX) is a commonly used chemotherapy drug that exerts adverse toxic effects on both kidney and germ cells. Exposure to MTX increases nitric oxide (NO) production, depleting CAT, superoxide dismutase (SOD), glutathione reductase (GR), glutathione peroxidase (GPx), and reduced GSH in testicular tissue [57]. Naringin can significantly counteract the above effects of MTX, suggesting that naringin may be a potential candidate for the toxic effects of MTX on reproduction. Similarly, naringin has a nephroprotective effect in methotrexate-induced nephrotoxicity in male rats, which involves significant increases in antioxidant enzymes and GSH levels. However, the nephroprotective molecular mechanism of naringin still needs to be further studied [58].

Cisplatin (CP) and aminoglycosides are effective anti-tumors or bactericidal drugs, and their application may cause hair cell (HCs) death, so there is a high probability of ototoxicity. In zebrafish animal models, naringin can play antioxidant, anti-apoptotic, proliferative, and HCs regeneration roles by downregulating the expression of genes related to apoptosis (P53 or Bcl-2, Bax, and Caspase-3) and pyroptosis (caspb and caspa or Caspase-1 and NLRP3), thereby achieving anti-ototoxic potential in vivo and in vitro [59].

Doxorubicin (DOX) is an anti-tumor antibiotic, and toxic side effects on the heart can lead to cardiomyocyte damage and irreversible heart failure, limiting its clinical application. In a constructed DOX-treated mouse model, naringin could inhibit the effects of DOX on apoptosis, inflammation, and oxidative stress in vitro experiments by promoting the expression of ECHS1, which is an essential reason for protecting mouse hearts from damage [60].

Oxaliplatin (OXL), a chemotherapy drug used for metastatic and other types of cancer, causes peripheral neuropathy with dose-limiting side effects. A study has revealed that naringin can reduce oxidative stress, inflammation, and apoptosis in OXL-induced sciatic nerve tissue. It significantly attenuates peripheral neuropathy and can be a novel protective agent in preventing OXL-induced peripheral neuropathy [61].

Naringin reduces the toxic effects of various clinical drugs mainly by reducing oxidative stress, inflammation, apoptosis, and pyroptosis, promoting cell proliferation and regeneration, and balancing the metabolism of nutrients (glucose and proteins), suggesting that naringin combined with other drugs may be a better clinical approach in treating diseases.

### 3.5 Inflammation and naringin

Inflammation is a protective response to environmental exposure, infection, tissue damage, and autoimmune induction, mainly manifested as redness, swelling, heat, pain, immune cell infiltration, and dysfunction [62]. However, severe and uncontrolled persistent inflammation is a pathological process with local or systemic tissue involvement that leads to inflammatory disease [63]. Inflammatory disorders share common physiologic and pathological features and are caused by excessive, abnormal, and sustained immune responses in immune cells and inflammatory responses in stromal cells [64,65]. Thus, early control of the development of abnormal immune reactions is a promising strategy for treating inflammatory diseases.

Peripherally induced inflammation promotes transient activation of TNF-α-mediated primer neural stem cells (NSCs), which can enhance the systemic inflammation alarm of adult NSCs through TNF-α receptor signaling and drive inflammation [66]. Accurate regulation of homeostasis in the inflammatory process is significant for treating inflammation-related diseases. Naringin is a natural anti-inflammatory antioxidant that actively participates in the body's immune response and maintains the integrity of the immune barrier. It can enhance autophagy flux by activating the AMPK/SIRT1 pathway, protecting human nuclear myelocytes from TNF-α-induced inflammation, oxidative stress, and loss of cellular homeostasis [67]. It is worth noting that inflammation and oxidative stress are two-way processes. Thus, limiting oxidative stress is also an effective way to control inflammatory diseases.

### 3.6 Naringin against neuroinflammatory diseases

Neuroinflammation is primarily caused by an immune response and long-term activation of the brain's glial cells (astrocytes and microglia). Usually, it is a crucial process in the pathogenesis of neurodegenerative diseases (NDDs) [64,68]. NDDs mainly include cerebral ischemia, brain injury, Alzheimer's disease (AD), and Parkinson's disease (PD). The pathophysiology of neurodegeneration is complex, and effective treatments for this disease are lacking. Thus, naringin extracted from medicinal plants or fruits shows promise in targeting multiple signaling pathways to treat different types of neuroinflammatory disorders.

#### 3.6.1 Naringin and Alzheimer's disease

Alzheimer's disease (AD) is the most common neurodegenerative disease, with the clinical manifestation of an individual's cognitive dysfunction and progression to dementia. The leading cause of AD is the accumulation of hyperphosphorylated tau protein, Amyloid-β (Aβ) plaques, and neurofibrillary tangles (NFTs) [69]. So far, studies have shown that estrogen and mediated cell signaling pathways have some neuroprotective effects on AD in the absence of effective and safe cures and drugs [70]. Unfortunately, long-term estrogen therapy can cause unpredictable side effects such as uterine cancer.

As a phytoestrogen, naringin has few harmful side effects, and it can bind to estrogen receptors (ERs) to initiate estrogen-like biological effects [71]. Studies have found that naringin stimulates the expression of ER β, activates the PI3K/AKT signaling pathway, and inhibits the activity of glycogen synthase kinase (GSK)-3β and phosphorylation of Tau protein [72]. As an alternative to long-term estrogen therapy, the application of naringin is a valuable research direction for anti-AD treatment. Naringin has a strong iron chelating ability, which can reduce the formation of amyloid plaques. Therefore, it can be used for neuroprotection and prevention of AD [73]. Meanwhile, naringin can also improve memory deficits and cognitive dysfunction by regulating multiple metabolic pathways and exerting neuroprotective effects in animal models of AD [74]. As an example, expression of the brain-derived neurotrophic factor (BDNF)/cAMP response element binding protein (CREB)/tropomyosin receptor kinase B (TrkB) signaling pathway plays a crucial role in improving learning and memory [75]. Aβ inhibits the expression of TrkB and CREB in the hippocampus. Still, naringin can enhance the presentation of BDNF, TrkB, and CREB in the long term and attenuate the activated cyclooxygenase-2 under inflammatory conditions, thereby compensating for the learning and memory deficits in the rat model of AD-like behavior [76]. In addition, naringin exerts therapeutic benefits in Aβ-induced rat animal models through the renin-angiotensin system. The mechanism of action is that naringin improves not only Aβ-induced cholinergic dysfunction but also reverses Aβ-induced apoptosis, maintains increased levels of mitochondrial calcium uniporter, and decreases the level of hemeoxygenase-1 in all brain regions, thereby countering amyloid β-induced brain cognitive deficits and mitochondrial toxicity in rats [77].

Furthermore, naringin can also reduce the expression of phosphorylated p-P38/P38 [78], one of the main proteins in the mitogen-activated protein kinase (MAPK) family, p38 MAPK, together with c-Jun N-terminal kinase (JNK), is the main pathway by which MAPK regulates cellular physiological responses [79]. When p38 MAPK is activated, it forms a stress MAPK signaling pathway, often triggered by environmental stress and cytokines to induce inflammation and accelerate apoptosis [80]. However, whether naringin exerts neuroprotective effects via the P38 MAPK pathway needs further verification.

At present, naringin has been shown to reduce the inflammatory response, oxidative stress, and endoplasmic reticulum stress by inhibiting the TLR4/NF-κB signaling pathway, thereby improving cognitive dysfunction and hippocampal histopathological injury in rats [81]. In summary, naringin can exert neuroprotective effects through a variety of mechanisms, including the BDNF/CREB)/TrkB signaling pathway, TLR4/NF-κB signaling pathway, renin-angiotensin system, amyloid β metabolism, Tau protein hyperphosphorylation, acetylcholinergic system, oxidative stress, and apoptosis. Of note, naringin primarily enhances long-term memory in animal models of AD by regulating autophagy, oxidative stress, and tau expression [82].

Due to the bitter taste of naringin, it is necessary to find alternative derivatives while retaining their functional advantages. Naringin dihydrochalcone (NDC), a natural naringin derivative widely used in food, has a sweet taste and a powerful antioxidant effect. The study has found that NDC can weaken Aβ deposition in the brain of AD mice and reduce the activated microglia and astrocytes around the plaque, inhibiting neuroinflammation [83].

#### 3.6.2 Naringin and Parkinson's disease

Parkinson's disease (PD) is also a chronic neurodegenerative disorder with degenerative death of dopaminergic neurons in the substantia nigra of the midbrain, followed by a decrease in dopamine levels in the striatum [84], characterized by bradykinesia, resting tremor, and muscle rigidity and postural gait disturbance. Despite extensive research, treatment options for the disease are limited and can only relieve symptoms rather than stop disease progression. Naringin is a flavonoid with neuroprotective activity, which can promote the production of glia-derived neurotrophic factor (GDNF) by dopaminergic neurons in the substantia nigra striatum, reduce the level of TNF-α in microglia, and then protect dopaminergic projection in the substantia nigra striatum in PD, playing an anti-inflammatory and preventive therapeutic role in brain dopaminergic neurons [85,86]. In line with this, Kim HD et al. further demonstrated that naringin does prevent dopaminergic neuronal degeneration but may not be sufficient to restore the substantia nigra striatal dopaminergic neuronal projection in a mouse model of PD [87]. In addition, there is growing evidence that the Nrf2-centric signaling pathway may be a critical pharmacological target for treating NDDs [88]. In animal models of rotenone-induced PD, naringin can exert neuroprotective activity on rotenone-induced toxicity in animals via the Nrf2-mediated pathway [89]. Therefore, it is speculated that naringin may be another option for future PD treatment management.

#### 3.6.3 Naringin and cerebral ischemia-reperfusion injury

Recently, in an in vitro cell experiment, naringin was found to prevent PC12 cell damage in an oxygen-glucose deprivation/reoxygenation (OGD/R) model by targeting NF-κB1 and regulating the HIF-1α/AKT/mTOR signaling pathway, providing new ideas for the treatment of cerebral ischemia-reperfusion injury (IRI) [90]. In addition, naringin can also attenuate cerebral IRI injury by inhibiting peroxynitrite-mediated mitochondrial autophagy [91]. It is worth noting that prolonged total cerebral ischemia or severe ischemia can cause cerebral infarction, while naringin can protect against cerebral infarction by inhibiting neuronal apoptosis and inflammation. For example, naringin could reduce apoptosis of rat hippocampal nerve cells and secretion of inflammatory factors such as TNF-α and IL-6 in rat animal models with cerebral infarction and further promote the expression of *p*-AKT protein in a concentration-dependent manner, thereby activating the PI3K/AKT pathway in neurons [92].

#### 3.6.4 Naringin and depression

In addition, naringin can mediate neurogenesis in adults by activating CREB signaling to combat depression [93]. In line with this, studies have found that flavonoid extract naringin can be recovered from agricultural residues with anxiolytic and antidepressant-like effects [94]. Therefore, naringin may have an excellent therapeutic potential for anxiety and depression.

It has been found that the leptin-Janus kinase (JAK)/signal transducer and activator of transcription (STAT) 3 signaling pathway is activated in the microglia of sleep-deprived patients. Naringin can promote microglial polarization to M2-type macrophages by inhibiting phosphorylation of the JAK/STAT3 signaling pathway, thereby promoting anti-inflammatory, tissue repair, extracellular matrix reconstruction, and exerting neuroprotective effects [95].

### 3.7 Naringin against cardiovascular inflammatory diseases

Cardiovascular disease (CVD) is a class of diseases of the heart or blood vessels that persist for a long time and harm human health. Hyperglycemia, hyperlipidemia, hypertension, obesity, heavy alcohol consumption, lack of exercise, smoking, inflammation, and clonal hematopoiesis are important risk factors for CVD [96,97]. These risk factors help initiate mechanisms such as arterial wall inflammation and oxidation, leading to the formation of fatty fibrous lesions over time [96]. Therefore, atherosclerosis is the pathological basis for the development of CVD.

#### 3.7.1 Naringin and atherosclerosis

Atherosclerosis (AS) is a chronic inflammatory vascular disease driven by risk factors, lipid metabolism disorders as the pathological basis of AS, early arterial lesions starting from the intima, initial deposition of lipoproteins in the vascular wall, endothelium activation and expression of chemokines and adhesion molecules leading to leukocyte recruitment and infiltration into the subendothelium, and subsequent accumulation of cholesterol and low-density lipoprotein (LDL) into macrophages in the arterial wall [98,99], which eventually leads to thickening and hardening of the artery wall, narrowing of the lumen of the vessel, and even blockage. Naringin has been shown to lower cholesterol and triglyceride (TG), promote lipolysis, and regulate fatty acid β oxidation in a dose-dependent manner [[100], [101], [102], [103], [104]]. Various studies involved in vitro and in vivo models have shown that naringin has a positive effect on inhibiting AS progression. Hypercholesterolemia is one of the risk factors for AS, and naringin reverses vascular dysfunction and oxidative stress in hypercholesterolemia rats by reducing iNOS, LOX-1, and NADPH oxidase subunit expression. This process also involves a decrease in ROS production, increasing NO bioavailability and improving rat aortic endothelial function [105]. In addition, studies have revealed that the main potential pathway for naringin to alleviate is the gut microbiota-liver-cholesterol axis, which promotes the synthesis of bile acids in cholesterol by inhibiting the FXR/FGF15 pathway and upregulating CYP7A1 [98]. Moreover, the results also show that naringin promotes reverse cholesterol transport by downregulating PCSK9/IDOL [106]. In addition to the mechanisms described above, the potential protective effects of naringin against AS can reverse apoptosis and inflammation triggered by oxidative LDL (ox-LDL) by inhibiting the YAP pathway [6]. In human umbilical vein endothelial cells (HUVECs), naringin restores endothelial barrier integrity by preventing VE-cadherin disassembly and F-actin remodeling, as well as down-regulation of pro-inflammatory factors such as IL-1β, IL-6, and IL-18 [6]. In conclusion, naringin has an apparent regulatory effect on AS pathological lipid metabolism and inflammatory state, which may attenuate the onset of AS to a certain extent.

#### 3.7.2 Naringin and diabetic cardiomyopathy

Diabetic cardiomyopathy (DCM) is the primary disease of people with diabetes and is characterized by diastolic dysfunction leading to heart failure and death. Unfortunately, even strict blood sugar control is not practical in preventing it. Naringin limits the increase in ROS production, reduces calpain activity, TNF-α, and IL-6 levels, and decreases NF-κB expression, thereby avoiding cardiomyopathy in type 2 diabetes (T2DM) mice [107].

#### 3.7.3 Naringin and diabetic cardiac autonomic neuropathy

Diabetic cardiac autonomic neuropathy (DCAN) is one of the most common complications associated with diabetes. In cases of diabetes, P2Y14 is significantly overexpressed, releasing inflammatory factors (IL-1β) in large quantities, forming gap connections between nerve cells, which promote DCAN, which can lead to cardiac damage [108]. Naringin reduces the expression of P2Y14 receptors and inflammatory factors and restores the function of the antioxidant GPX4/NRF2 pathway, thereby effectively alleviating DCAN [108].

#### 3.7.4 Naringin and cardiac hypertrophy

Cardiomyocytes exit the cell cycle and undergo terminal differentiation shortly after birth, so adult cardiac hypertrophy increases in the size of a single cardiomyocyte [109]. Hypertrophy is initially an adaptive response of cardiomyocytes to stimuli to maintain cardiac output during harmful stimuli, a process known as physiological hypertrophy. However, sustained stimulation in the later stages can cause myocardial decompensation. That is, pathological hypertrophy may lead to heart failure. Myocardial hypertrophy involves different signaling pathways related to regulation. ERK, a signaling molecule downstream of the MAPK pathway (also known as RAS-RAF-MEK-ERK), is a key player in the pathophysiology of cardiac hypertrophy and appears to play a beneficial role by preventing cell death and hypertrophy during chronic stress overload [110]. In addition, naringin improves hyper fructose-induced cardiac hypertrophy by regulating the AMPK-mTOR signaling axis to reduce cardiomyocyte hypertrophy [111].

Leptin is a product of the obesity gene and plays a role in the pro-inflammatory immune response, angiogenesis, cardiomyocyte hypertrophy, and lipolysis [112,113]. Chen J et al. demonstrated that in cardioblasts (H9c2 cells), leptin induces rat cardiomyocyte hypertrophy through p38/MAPK activation. In contrast, naringin protects heart cells from high glucose-induced damage by inhibiting the leptin-induced p38/MAPK pathway [114]. In subsequent studies, naringin can attenuate high glucose-induced leptin expression and mitigate high glucose-induced cardiomyocyte damage and inflammatory responses by inhibiting the JAK2/STAT3 pathway in H9c2 cardiomyocytes [115].

#### 3.7.5 Naringin and myocardial ischemia-reperfusion injury

Ischemia-reperfusion injury is one of the significant risk factors associated with cardiovascular morbidity and mortality. Naringin can be used as an emerging therapeutic drug with the potential to treat ischemic diseases. Endothelial progenitor cells (EPCs) can be involved in neovascularization in vivo. Activation of the CXCL12/CXCR4 axis by the PI3K/AKT pathway enhances endothelial progenitor cell proliferation [116], exhibiting a positive role in treating myocardial IRI. Naringin can significantly promote the phosphorylation of Akt, so naringin alleviates myocardial IRI in rats by promoting apoptosis, oxidative stress, and autophagy inhibition mediated by the PI3K/Akt pathway [117]. In addition, naringin can also inhibit cardiomyocyte apoptosis, inflammatory response, and oxidative stress by reducing the expression of lysis caspase 3 protein, IL-23, IL-6, and TNF-α, SOD, which in turn has a particular alleviating effect on myocardial IRI in rats [118]. In a recent study, naringin can also significantly upregulate miR-126 expression, and miR-126 can bind to GSK-3β and downregulate its expression, thereby increasing β-catenin activity in cardiomyocytes to reduce myocardial IRI [119].

### 3.8 Naringin against inflammatory bowel diseases

Several recent studies have demonstrated that naringin can significantly improve inflammatory bowel diseases, including ulcerative colitis (UC) and sepsis-induced intestinal damage, by inhibiting inflammatory responses and regulating intestinal flora homeostasis [[120], [121], [122], [123]]. Among them, naringin inhibits the intestinal inflammatory response involving several pathways and signaling molecules.

#### 3.8.1 Naringin and ulcerative colitis

Peroxisome proliferator-activated receptor γ (PPARγ) is an essential member of the nuclear receptor superfamily, which can inhibit the expression of pro-inflammatory genes and is considered a necessary regulator of inflammation [124]. Therefore, modulating PPARγ and PPARγ-related pathways has great promise for treating UC. As an example, in dextran sodium sulfate (DSS)-induced mouse UC animal models, naringin significantly alleviated DSS-induced disease activity index (DAI), colon length shortening, and colonic pathological damage, and correspondingly reduced tissue and serum secretion of inflammatory cytokines (TNF-α, IL-6, and IL-1β) [125]. On the one hand, the expression of PPAR-γ in DSS-induced colitis is significantly reduced, which may lead to an increase in the expression of the nuclear transcription factor NF-κB. However, naringin can activate DSS-induced PPARγ and subsequently inhibit NF-κB activation, reduce inflammatory cytokines, and inhibit inflammation [126]. Furthermore, the symptomatic process of naringin in colitis relief can be counteracted by the PPAR-γ inhibitor BADGE, suggesting that PPAR-γ may be the target of the therapeutic effect of naringin-induced colitis [126]. On the other hand, the MAPK pathway in DSS-induced mice is also significantly activated, and naringin significantly inhibits p38, ERK, and JNK phosphorylation levels, thereby inhibiting the MAPK signaling pathway and reducing NLRP3 inflammasome activation [9]. Therefore, naringin can inhibit NF-κB and MAPK signaling pathways, reduce the production of pro-inflammatory factors, and have a specific remission effect on DSS-induced UC in mice. Similarly, in a recent study, naringin was shown to mitigate TNBS-induced colitis in rats by reducing pro-inflammatory cytokines and improving antioxidant status [127]. In addition, naringin may lessen the severity of colitis by inhibiting pro-inflammatory mediators (GM-CSF/M-CSF, IL-6, and TNF-α) and the NF-κB/IL-6/STAT3 signaling pathway in colorectal tumor pathogenesis [128]. In conclusion, NG may be a compelling candidate for treating UC patients.

#### 3.8.2 Naringin and sepsis-induced intestinal injury

Macrophages are an essential component of the innate immune system and contribute significantly to the inflammatory response. Once activated, they can polarize into two different phenotypes, classically activated macrophages (M1) and alternatively activated macrophages (M2), and the nuclear receptor PPARγ can drive macrophage phenotypic changes [129]. In animal models of intestinal injury in sepsis, naringin therapy significantly inhibited M1 macrophage polarization and stimulated M2 macrophage polarization by activating the PPARγ/miR-21 axis to inhibit STAT1 signaling [130], alleviating sepsis-induced intestinal injury. In addition, naringin can improve impaired intestinal permeability and inhibit the release of pro-inflammatory factors (TNF-α and IL-6), attenuate MLC phosphorylation and NF-κB activation through the RhoA/ROCK pathway in vivo and in vitro, increase the expression of tight junction proteins ZO-1 and claudin-1, protecting against sepsis-induced intestinal injury [131].

### 3.9 Naringin against inflammatory lung disease

The anti-inflammatory effect of naringin under lung injury is mediated by inhibition of NF-κB signaling, and decreased levels of anti-inflammatory cytokines (IL-6, TNF-α, IL-1 β) can be detected [124]. At the same time, naringin treatment also significantly reduced the expression levels of p-p38 and *p*-JNK proteins, inhibited the MAPK signaling pathway, and blocked apoptosis of lung cells [132]. In mouse animal models of acute lung injury, naringin can reduce the protein expression of NF-κB, STAT3, and COX-2 and reduce the expression level of pro-inflammatory mediators, upregulating anti-inflammatory mediator levels [133]. Naringin has been found to reduce airway inflammation and lung permeability by upregulating Aquaporin1 (AQP1) expression in mouse animal models of chronic obstructive pulmonary disease (COPD) [15]. Nrf2 is an important mediator to protect cells from oxidative stress and can downregulate the expression of the NLRP3 inflammasome for anti-inflammatory effects [134]. During lung aging, naringin can induce SIRT1 to activate the Nrf2/NQO1 pathway to inhibit oxidative stress and promote the sirt1-FOXO1 pathway to induce autophagy [135]. Thus, delaying cellular aging can prolong the life span of experimental animals.

Naringin may act as an antiasthmatic drug to relax tracheal smooth muscle in rats, mainly by opening Ca2+-activated K+ channels to relax tracheal smooth muscle, mediating plasma membrane hyperpolarization and reducing Ca2+ influx [136]. At the same time, naringin is a non-toxic bitter substance of plant origin that promotes the proliferation of cultured human airway epithelial cells and activates bitter receptors (TAS2Rs), thereby initiating the relaxation of airway smooth muscle cells (ASMCs) [137]. Therefore, as a novel bronchodilator, naringin has great potential in treating asthma.

### 3.10 Naringin against liver inflammation

#### 3.10.1 Naringin and nonalcoholic fatty liver disease

Nonalcoholic fatty liver disease (NAFLD) is a disorder of lipid metabolism caused by various factors other than alcohol and other definite liver damage, and excessive fructose intake leads to hepatic lipid accumulation through increased TG synthesis [138]. Risk factors for nonalcoholic liver disease include obesity, diabetes mellitus, insulin resistance, hyperlipidemia, nutrition, and genetic factors [139]. In addition, there is emerging evidence that gut bacteria are strongly associated with the development of NAFLD [140]. Currently, clinical guidelines recommend a weight loss target of 7–10% to improve the character of NAFLD [141]. The lipid-lowering effect of naringin can effectively reduce NALFD induced by a high-fat diet, attenuate body weight, and decrease liver lipid accumulation in mice [142]. Naringin can improve intestinal bacterial dysbiosis and liver resistance to oxidative stress and inflammation [143]. In a novel tissue engineering fatty liver model, naringin can downregulate CD36, acetyl-CoA carboxylase, and fatty acid synthetase, increase PPAR-α, thereby reducing fatty acid uptake and new fat production, and increase fatty acid oxidation to improve lipid metabolism disorders in the liver [144]. Qu Zhi Ke (the peel of Changshan pomelo containing naringin) significantly inhibits systemic and intrahepatic inflammation by inhibiting IL-1β, IL-6, IL-12, TNF-α, and IFN-γ, thereby inhibiting NF-κB signaling pathways in the liver driven by IL-1, TNF-α, and other inflammatory factors [145]. In addition to NF-κB signaling, Qu Zhi Ke can also inhibit MAPK signaling by inhibiting the phosphorylation of ERK and p38 [145]. Among them, P38 is a member of the MAPK subfamily, which can induce the secretion of pro-inflammatory factors (CXCL2, IL-1β, CXCL10, and IL-6) by M1 macrophages to promote the development of steatohepatitis [146]. Therefore, hepatoprotective and anti-inflammatory effects of naringin in NAFLD lie in inhibiting NF-κB and MAPK signaling. In line with this, naringin has been shown to reverse the upregulation of NF-κB and TNF-α expression in rat liver in fructose-induced rat NAFLD animal models and target activation of antioxidant mediator Nrf2/HO-1 pathways to block fructose-induced NAFLD progression in rats [147]. As NAFLD progresses, it may become a common chronic liver disease, and liver fibrosis develops. Liver fibrosis is mainly the activation of hepatic stellate cells, which in turn occurs excessive accumulation of extracellular matrix and collagen deposits, which can lead to the development of cirrhosis and even liver cancer [148,149]. Naringin has anti-fibrotic properties with few side effects and is also a promising source in treating liver fibrosis.

#### 3.10.2 Naringin and diabetes-induced hepatitis

Naringin improves T2DM-induced steatohepatitis by inhibiting RAGE/NF-κB-mediated mitochondrial apoptosis [150]. However, in streptozotocin-induced rat models of type I diabetes, apoptosis of two pathways increases in diabetic states: protein expression of Fas/FasL/caspase-3 and an increase in Bax/Bcl-2 ratio and increased oxidative stress in the liver in diabetic rats shows increased levels of NF-κB, Cox-2, and IL-6. At the same time, NF-κB promotes iNOS gene expression and subsequent NO formation, increasing nitrosylation of proteins [151]. However, naringin can significantly eliminate these effects, highlighting its antioxidant, anti-nitrifying, and anti-inflammatory properties.

### 3.11 Naringin against renal inflammation

#### 3.11.1 Naringin and renal interstitial fibrosis

Renal interstitial fibrosis is typical of all progressive kidney diseases. The presence of profibrotic factors and inflammation often accompanies the fibrotic process. TGF-β1 is considered a key mediator of tissue fibrosis, and inhibiting TGF-β subtype TGF-β1 or its downstream signaling pathway can significantly limit renal fibrosis [152]. Smad2 and Smad3 are the two major downstream regulators that promote TGF-β1-mediated tissue fibrosis, which can bind to Smad4 to form complexes that trigger the expression of fibrotic genes. In contrast, Smad7, as a negative feedback regulator of the TGF-β1/Smad pathway, prevents TGF-β1-mediated fibrosis [153]. In rat models and fibrotic cell models of renal interstitial fibrosis, naringin can significantly reduce gene expression levels of α-smooth muscle actin (α-SMA), collagen 1 (COL1A1), collagen 3 (COL3A1), IL-1β, IL-6, and TNF-α, thereby reducing renal inflammation and fibrosis [154]. Notably, naringin reduces the expression of Smad2/3 and Smad4 phosphorylation and antagonizes renal interstitial fibrosis by inhibiting the TGF-β/Smad pathway.

#### 3.11.2 Naringin and diabetic nephropathy

In rat in vitro cultured mesangial cells, naringin can regulate the NLRP3-caspase-1-IL-1β/IL-18 signaling pathway to inhibit NLRP3 inflammasomes, reduce the expression of inflammatory factors and then improve diabetic nephropathy by exerting anti-inflammatory effects [155]. In addition, studies have found that naringin can prevent diabetic nephropathy in rats by blocking oxidative stress and mitochondrial dysfunction [156].

#### 3.11.3 Naringin and renal ischemia-reperfusion injury

In one study, naringin was found to benefit renal protection from renal IRI in rats by downregulating microRNA-10a, caspase-3, and Bax and upregulating the expression of Bcl-2 in renal tissue [157]. In addition, in rat animal models of renal IRI, naringin can improve the expression of SOD and Nrf-2 after renal IRI injury, enhance antioxidant activity, and thus have a clear protective role against renal and distal myocardial injury [158].

### 3.12 Naringin against skin inflammation

The skin is the body's biological barrier against external environmental stimuli and stress, and inflammation or wounds occur when its physiological structure is destroyed. Skin wound injuries are common and can lead to severe complications if not treated properly. Naringin can accelerate wound healing by upregulating the expression of growth factors (VEGF-A, B, C, and VEGF-R3) [159]. In addition, inhibiting NF-κB and COX-2 by topical application of naringin hydrogels enhances the repair and regeneration of deep dermal wounds [160]. Naringin has potent anti-inflammatory and antioxidant activities, not only able to downregulate gene expression of the inflammatory mediator (TNF-α) and apoptosis (Bax) but also to increase levels of SOD and GSH [161]. The combination of naringin/sericin can downregulate pro-inflammatory cytokines (including IL-6, IL-12p40, IL-23, and TNF-α) to help human peripheral blood mononuclear cells (hPBMCs) in patients with psoriasis exert anti-inflammatory effects [162]. Chronic inflammation of skin disorders to a later stage may develop into skin fibrosis. Naringin may play a protective role against fibrosis in skin tissue in vivo by reducing collagen production and lowering levels of fibrosis-related genes [163]. Similarly, naringin inhibits the phosphorylation of Akt and Akt downstream proteins in fibroblasts, thereby inhibiting the development of proliferative scars [164].

### 3.13 Naringin and joint inflammation

Rheumatoid arthritis (RA) is a chronic autoimmune disease characterized by persistent synovial hyperplasia and progressive erosion of articular cartilage. Potential mechanisms for the therapeutic effects of naringin in RA are mainly through the PI3K/Akt and MAPK/ERK signaling pathways that inhibit the production of inflammatory cytokines and matrix metalloproteinases (MMP), promoting apoptosis in RA fibroblast-like synoviocytes [165]. Similarly, naringin prevents cartilage destruction in osteoarthritis by inhibiting the NF-κB signaling pathway [166]. Furthermore, naringin can prevent steroid-induced avascular necrosis of the femoral head by upregulating PPARγ and activating the Notch signaling pathway [167]. In addition, naringin increases the expression of TGF-β2, TGF-β3, and Sox-9 in cartilage defects through the TGF-β/ALK5/Smad2/3 signaling pathway, thereby achieving good results in joint cartilage quality repair at the defect site [168], so naringin has good application potential in osteochondral tissue engineering.

### 3.14 Safety assessment of naringin

Safety assessment plays a vital role in researching and developing natural products. Wang Y et al. evaluated the potential toxicological effects of naringin on the reproductive system of SD rats, and the results showed that naringin gavage had minimal or no effect on clinical signs, reproductive performance, estrous cycle, spermatogenesis evaluation, gross pathology, and histopathology in rats [169]. Naringin's no-observed adverse effect level (NOAEL) on fertility and early embryonic development in rats was at least 1250 mg/kg/d [170]. In addition, Li P et al. compared the liver and kidney serological indices and toxicological changes in tissues and organs of rats given naringin with those of normal rats and found that naringin did not induce significant hepatotoxicity and nephrotoxicity [170,171].

Subsequently, the team further observed the morbidity and mortality of Beagles after continuous oral administration of naringin for 3 and 6 months, resulting in no toxicology-related events [172]. In vitro experiments also verified that naringin was cytotoxic to cancer cells at low concentrations but not healthy cells [173]. At the same time, naringin did not cause significant DNA damage at non-cytotoxic concentrations but was protective against DNA damage in healthy cells. These in vivo and in vitro results indicate that naringin has a good safety profile.

However, Ranawat's team found that naringin was able to reduce the number of apoptotic germ cells as well as sperm count and viability in mice, thereby causing damaging effects on testicular tissue [174]. This is mainly because that although naringin is a potent antioxidant, it may also be a pro-oxidant [174]. Limited toxicokinetic data are available for naringin. The toxicity of 22 polyphenol-rich compounds was assessed using a high-content screening analysis. The early and late toxicity of naringin on cells was further observed by observing the effects of naringin on the mitochondrial membrane potential, cell membrane integrity, and nuclear size of the respective cells of five cell lines (HepG2, Caco-2, A549, HMEC-1, and 3T3), which confirmed that naringin is the least toxic phenolic compound [175]. Meanwhile, the total drug concentration detected 24 h after oral naringin administration in rats was significantly reduced, suggesting that naringin does not accumulate excessively in the body after a few days of administration [176,177]. This provides a reference for further research on naringin in human clinical studies.

Currently, studies on the adverse effects of naringin have focused more on animal studies, and few studies have documented adverse effects in humans from naringin-derived foods. However, Ortiz-Andrade et al. demonstrated through in vitro and in vivo experiments and computerized methods that naringin is relatively safe for human use, virtually non-toxic, and valuable for drug development [178]. In addition, studies have demonstrated that no alterations in serum hepatic or renal parameters and no significant adverse events (pruritus, vomiting, diarrhea, etc.) have been observed in healthy adults who ingested doses of 150–900 mg of citrus extract (28 % naringenin and 8.5 % naringin) [179]. At these doses, naringin metabolites were in circulation and cleared within 24 h. Meanwhile, naringenin (at 8 μM in blood and the appropriate dose) increased the thermogenic genes of primary human adipocytes, so it is suggested that a twice-daily intake of 300 mg of citrus extract would benefit humans [179]. However, the main component of citrus extract in this trial was naringenin rather than naringin, so more clinical trials may be needed to confirm naringin's safety in its pure form in humans.

### 3.15 Naringin clinical trial data

Naringin has been approved by the China Food and Drug Administration for clinical trials as a new drug because of its cough suppressant, phlegm, and low toxicity characteristics [180]. Current clinical trials of naringin/naringenin have focused on significant hypoglycemic, hypolipidemic, hypotensive [181], anti-inflammatory, and anti-oxidative stress efficacy, as well as the potential to affect the bioavailability of other medications in clinical applications (Table 1). However, these clinical trial models have focused more on the therapeutic effects of naringin in patients with different types of dyslipidemia. In addition, It is important to note that naringin should be avoided with honey, as honey significantly reduces the absorption of naringin/naringenin in humans [182]. Therefore, future clinical validation of naringin in other disease models must be supplemented.

**Table 1:** Clinical trials on the efficacy of naringin, naringenin, and its enriched food sources.

Model	Dose	Mechanism	Key findings	Ref.
Hypercholesterolemic subjects	Naringin, 400mg/capsule/day	Decreased plasma total cholesterol (TC), LDL, and Apo B; increased erythrocyte SOD and CAT activity	Lowering blood lipid, antioxidant	[183]
Patients with moderate hypercholesterolemia	Naringin, 500mg/capsule/day	TC and LDL cholesterol (LDL-C) concentrations are not reduced	Naringin does not lower blood lipids in patients with moderately high cholesterol	[184]
Adult patients with dyslipidemia	Naringin. 450 mg/day	TC, LDL, and TG decreased; lipocalin increased slightly	Reducing weight in patients with dyslipidemia	[102]
Prediabetes patients	Citrus Flavonoid Supplement (4 % Naringin). 200, 400, or 800 mg/day for 12 weeks	Decreased fasting glucose, HOMA-IR, HbA1c, glucagon, C-peptide, hsCRP, IL-6, and TNF-α; increased GLP-1, lipocalin in the blood.	Anti-inflammatory, anti-hyperglycemic and antioxidant	[185]
Patients with T2DM and mixed hyperlipidemia	650 mg of the BPF powder (BPF, main ingredient naringin)	LDL particles (LDL-P), TC, LDL-C, TG, blood glucose reduction	Lowering of blood glucose and blood lipids, complementary treatment of cardiometabolic disorders	[186,187]
T2DM	Mediterranean Diet (naringenin, naringin)	Decreased IL-6, oxidative stress marker 8-hydroxy-2′-deoxyguanosine (8-OHdG)	Reducing Inflammation in T2DM	[188]
Participants with abdominal obesity/dyslipidemia	1240 mg/day total polyphenols (naringin, naringenin)	Increases fatty acid oxidation in the liver, reduces inflammation by inhibiting nuclear factor κ-light chain enhancers in activated B cells, increases lipocalin	Blood pressure lowering, diet program to treat NAFLD.	[189]
older adults experiencing subjective cognitive decline (SCD)	400 mg Citrus Peel Extract (naringenin 3 mg)	IL-10, IL1-Ra, soluble TNF receptor I, soluble TNF receptor II increased; CXCL8/IL-8, IL-1β, IL-6, IL-17, TNF-α, IP10, MIP-1α, CCL2/MCP-1, CCL5/RANTES, IL-18, NO, thiobarbituric acid reactive substances, SOD decreased	Improves immediate memory, visuospatial/structural, language, attention, and delayed memory; reduces oxidative stress and neuronal damage and anti-inflammatory effects	[190]
Healthy volunteers	Felodipine with 250 mL grapefruit juice	Significantly inhibited CYP3A4 activity	Peak concentrations were higher, but half-life was unchanged. Grapefruit juice significantly increased the bioavailability of felodipine	[[191], [192], [193]]
Healthy volunteers	aliskiren with 300 mL grapefruit juice	Naringin inhibits human intestinal organic anion transporting polypeptide (OATP) 1A2	Reducing exposure to aliskiren	[194]
Healthy volunteers	Fexofenadine with 300 ml grapefruit juice	Naringin inhibits the OATP1A2 part of the intestine	Reduces the bioavailability of oral fexofenadine	[195]
Healthy volunteers	talinolol and 1050 mg of naringin	The high dose may inhibit the efflux transporter protein P-glycoprotein (P-gp), counteracting uptake inhibition.	Short-term high-dose supplementation with naringin does not affect the pharmacokinetics of talinolol.	[196]
Healthy volunteers	Nisoldipine coated tablets with 250 ml water, 250 ml grapefruit juice		Increasing the maximum concentration of nisoldipine and decreasing the time to reach the maximum concentration	[197]

### 3.16 Future prospects

Naringin has a low cost, comprehensive efficacy, and a long consumption history. It is widely used in basic and applied research on various inflammatory damages, showing significant anti-inflammatory and antioxidant effects [5,10]. The latest study also demonstrated the protective effect of naringin on various system cells (Cardiac cells, Endothelial progenitor stem cells, Human amniotic fluid-derived stem cells, Human periodontal ligament stem cells, Bone marrow stem cells, neuronal cells) [198], highlighting the critical role of the Nrf2 pathway. In this review, we systematically explored the molecular mechanism of naringin in improving inflammatory diseases in nervous, cardiovascular, bone, and other tissues and found that naringin mainly plays an essential role in regulating the immune microenvironment and the recovery of metabolic disorders. However, the analysis of the pathway mechanism of naringin on the therapeutic effect of inflammatory diseases has yet to form a systematic and comprehensive theory. Hence, an in-depth exploration of its pharmacological effects and molecular mechanisms is still the focus and hotspot of future research. It is worth noting that the metabolic, toxicity, and safety evaluation data of naringin clinical trials still need to be completed and further supplemented.

## 4 Conclusion

Naringin is an active pharmaceutical component rich in flavonoids in citrus plants, which has good anti-inflammatory, antioxidant, anti-apoptotic, anti-tissue fibrosis, and hypolipidemic effects. Given the poor bioavailability and low water solubility of naringin, a new drug carrier encapsulation of naringin can improve its pharmacological properties and pharmacokinetics, thereby improving the utilization rate of naringin in vivo. In in vitro and in vitro experiments, naringin can exert powerful anti-inflammatory and antioxidant properties through various molecular mechanisms and signaling pathways, which have good alleviating effects on various inflammatory diseases. In addition, naringin treatment has few adverse effects and can reduce the toxicity of other drug treatments at the relevant doses, thereby reducing the toxic side effects of drugs. Therefore, after further research, naringin may be a promising candidate for the future treatment of novel and safe inflammatory diseases. However, the current bioactive role of naringin in inflammatory diseases relies heavily on animal models and in vitro experiments, and more clinical trials are needed to validate its beneficial role in human health further.

## Funding

This study was supported by the 10.13039/501100001809National Natural Science Foundation of China (No. 81873163), the 10.13039/501100007129Key Project of Natural Science Foundation of Shandong Province (ZR2020KC024), and the 10.13039/501100007129Natural Science Foundation of Shandong Province (ZR2017LH042).

National Natural Science Foundation of China, China; Key Project of Natural Science Foundation of Shandong Province, China; Natural Science Foundation of Shandong Province, China?

## Ethics declarations

Review and/or approval by an ethics committee was not needed for this study as this is a review.

Informed consent was not required for this study as this is a review.

## Data availability statement

No data was used for the research described in the article.

## CRediT authorship contribution statement

**Yuan Peng:** Conceptualization, Writing – original draft. **Ruyi Qu:** Investigation, Methodology. **Shuqin Xu:** Visualization. **Hongsheng Bi:** Resources, Supervision. **Dadong Guo:** Conceptualization, Funding acquisition, Writing – review & editing.

## Declaration of competing interest

The authors declare that they have no known competing financial interests or personal relationships that could have appeared to influence the work reported in this paper.
